# Supplementary material for: Role of Water Models in Simulations of Ion Conduction in Potassium Channels
Source: J Chem Theory Comput. 2026 Jan 8;22(2):1177–86. doi: 10.1021/acs.jctc.5c01787 (PMC12854744; doi:10.1021/acs.jctc.5c01787)
Supplement: Supplementary file 1 [file ct5c01787_si_001.pdf]

Supporting Information

Role of Water Models in Simulations of Ion Conduction in Potassium Channels

Stefano Bosio,[1,2], Diego Gazzoni[3], Carmen Domene[4], Matteo Masetti[1], and Simone Furini[3*]*

[1]Department of Pharmacy and Biotechnology, Alma Mater Studiorum – University of Bologna, via Gobetti 87, 40129 Bologna, ITALY
[2]Computational & Chemical Biology, Fondazione Istituto Italiano di Tecnologia, via Morego 30, 16163 Genoa, Italy, ITALY
[3]Department of Electrical, Electronic, and Information Engineering "Guglielmo Marconi", Alma Mater Studiorum – University of Bologna, via dell'Università 50, 47521 Cesena (FC), ITALY
[4]Department of Chemistry, University of Bath, Claverton Down, Bath, BA2 7AY, UK

Table S1. List of MD simulations and corresponding conduction events. Conduction events were considered as soft knock-on when a water molecule moves across the SF.

Channel	Water model	Membrane Potential [mV]	MD simulations	Hard knock-on conduction events	Soft knock-on conduction events
KcsA	TIP3P	100	7 x 4.0 μs	60	0
		200	8 x 4.0 μs	346	0
		400	8 x 1.0 μs	436	0
KcsA	OPC	100	4 x 3.0 μs and 4 x 2.0 μs	16	11
		200	4 x 3.0 μs and 4 x 2.0 μs	52	10
		400	8 x 2.0 μs	234	42
MthK	TIP3P	100	8 x 2.0 μs	27	0
		200	4 x 2.0 μs and 4 x 1.0 μs	150	0
		400	4 x 1.5 μs and 4 x 1.0 μs	474	0
MthK	OPC	100	8 x 2 μs	34	4
		200	4 x 3.0 μs and 4 x 2.0 μs	113	6
		400	8 x 2.0 μs	173	37

Table S2. Regions used to discretize the MD trajectories of KcsA. Ions and water molecules were considered inside S0-S4, and C, when within these boundaries. Extracellular and Intracellular boundaries were computed as centre of mass of the corresponding group of atoms.

	Extracellular Boundary	Intracellular Boundary	Maximum distance from the channel axis [Å]
S0	backbone oxygens of Gly79	backbone oxygens of Tyr78	4
S1	backbone oxygens of Tyr78	backbone oxygens of Gly77	4
S2	backbone oxygens of Gly77	backbone oxygens of Val76	4
S3	backbone oxygens of Val76	backbone oxygens of Thr75	4
S4	backbone oxygens of Thr75	hydroxyl oxygens of Thr75	4
C	hydroxyl oxygens of Thr75	residues Thr107	8

Table S3. Regions used to discretize the MD trajectories of MthK. Ions and water molecules were considered inside S0-S4, and C, when within these boundaries. Extracellular and Intracellular boundaries were computed as centre of mass of the corresponding group of atoms.

	Extracellular Boundary	Intracellular Boundary	Maximum distance from the channel axis [Å]
S0	backbone oxygens of Gly63	backbone oxygens of Tyr62	4
S1	backbone oxygens of Tyr62	backbone oxygens of Gly61	4
S2	backbone oxygens of Gly61	backbone oxygens of Val60	4
S3	backbone oxygens of Val60	backbone oxygens of Thr59	4
S4	backbone oxygens of Thr59	hydroxyl oxygens of Thr59	4
C	hydroxyl oxygens of Thr59	residues Glu92	8

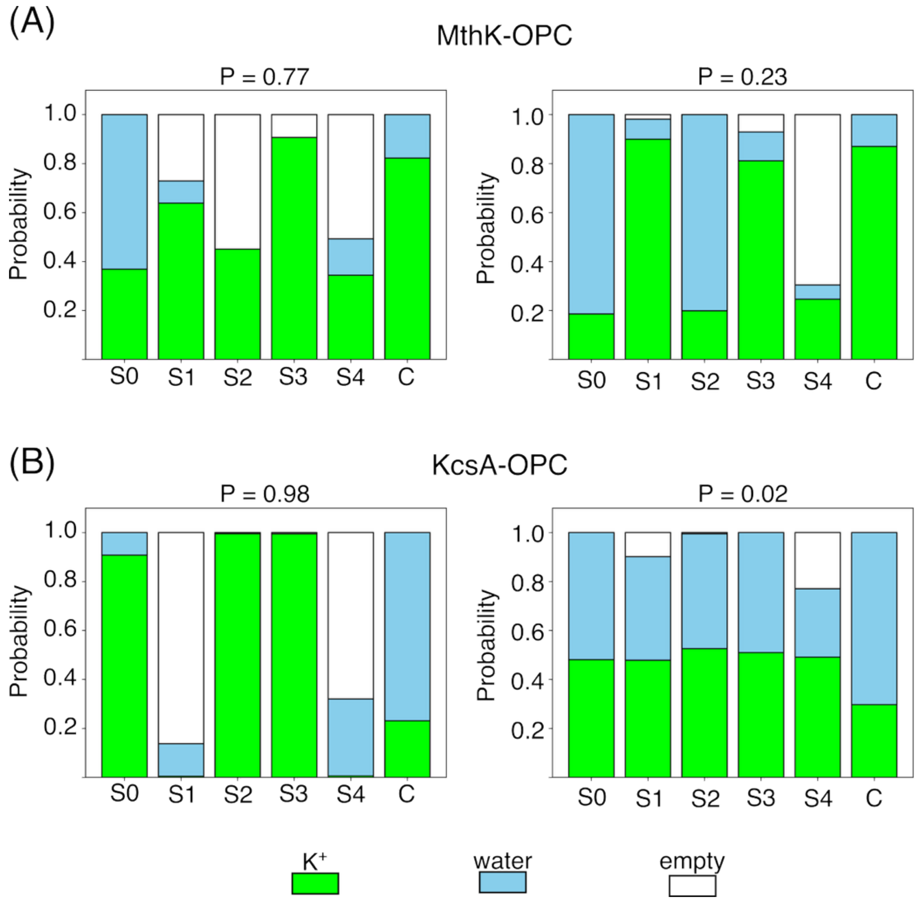

Figure S1. Average state of the SF in a two state models that separate slowest converting microstates in MthK (A) and KcsA (B) at 100 mV. The two macrostates were defined according to the sign of their projection along the eigenvector of the MSM corresponding to the slowest timescale. The average state of the SF was computed as the weighted average of the probability of the binding sites being empty, occupied by water, or occupied by potassium ions, respectively in the two clusters. The cumulative probability of the two clusters is reported at the top. Data refers to the MSM estimated at sampling period of 1 ns for simulations of MthK/KcsA with the OPC water model at membrane potential equal to 100 mV.

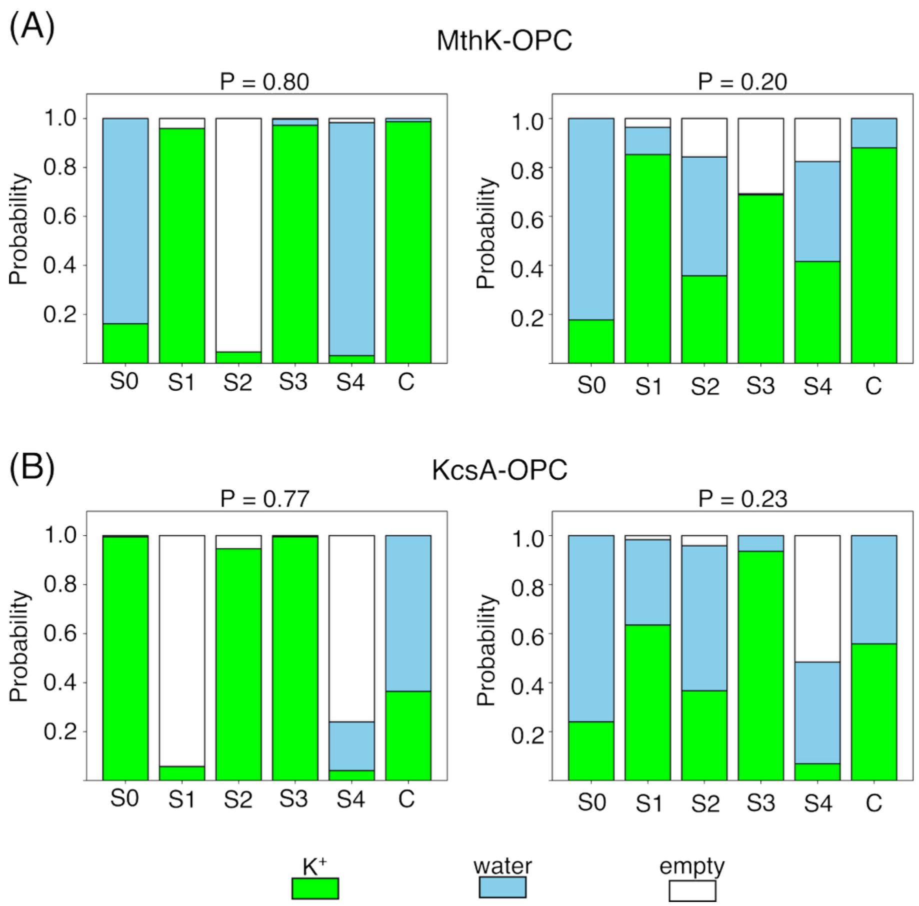

Figure S2. Average state of the SF in a two state models that separate slowest converting microstates in MthK (A) and KcsA (B) at 400 mV. The two macrostates were defined according to the sign of their projection along the eigenvector of the MSM corresponding to the slowest timescale. The average state of the SF was computed as the weighted average of the probability of the binding sites being empty, occupied by water, or occupied by potassium ions, respectively in the two clusters. The cumulative probability of the two clusters is reported at the top. Data refers to the MSM estimated at sampling period of 1 ns for simulations of MthK/KcsA with the OPC water model at membrane potential equal to 400 mV.

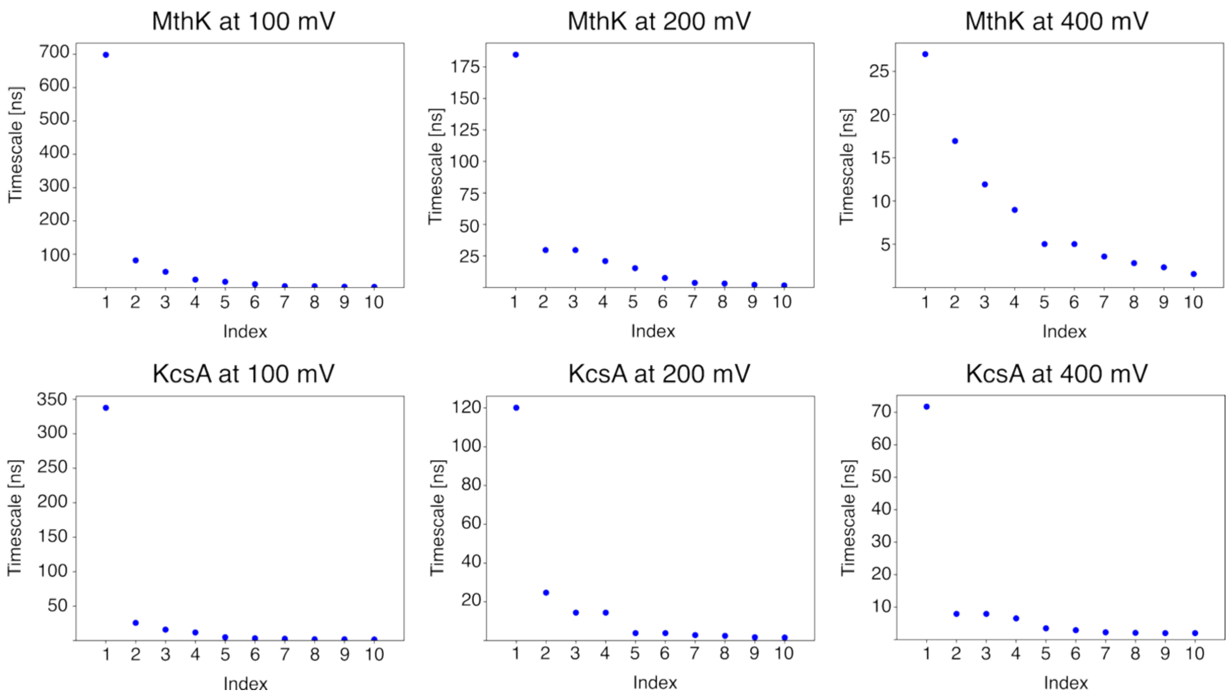

Figure S3. Relaxation times estimated from the transition matrix of the MSMs. The first 10 higher timescales are shown for both channels and all membrane potentials, as estimated from transition matrixes obtained with a sampling time equal to 1 ns.

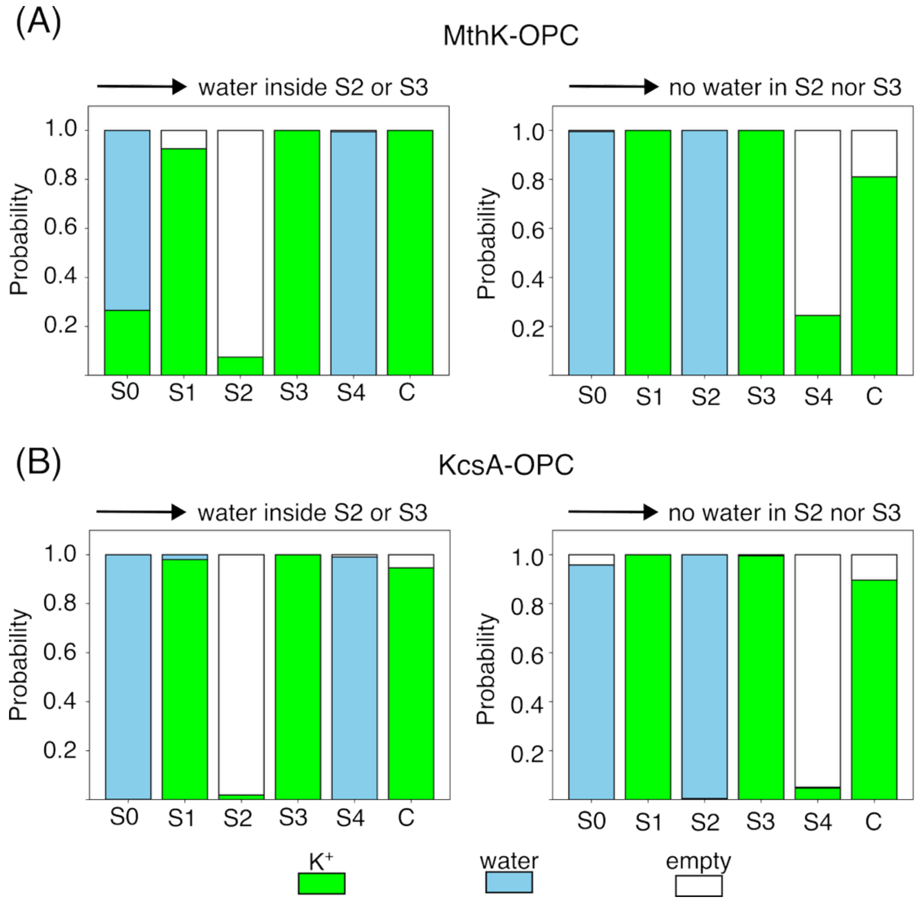

Figure S4. Average state of the SF that precedes the entrance of water molecules in S2 or S3 (left) or the depletion of S2 and S3 from water molecules (right). The average state of the SF that leads to the entrance of water molecules in S2 or S3 was calculated considering all the microscopic states of the MSM with no water in S2 nor S3 (*source*) that are connected to any microscopic state with water molecules in S2 or S2 (*sink*). Then, the weighted average of the probability of the binding site being empty, occupied by water, or occupied by potassium ions were calculated. The same method, switching *source* with *sink*, was used to calculate the average state of the SF that leads to the depletion of S2 and S3 from water molecules. Data refers to simulations of the MthK/KcsA channel with the OPC water model at a membrane potential of 100 mV.

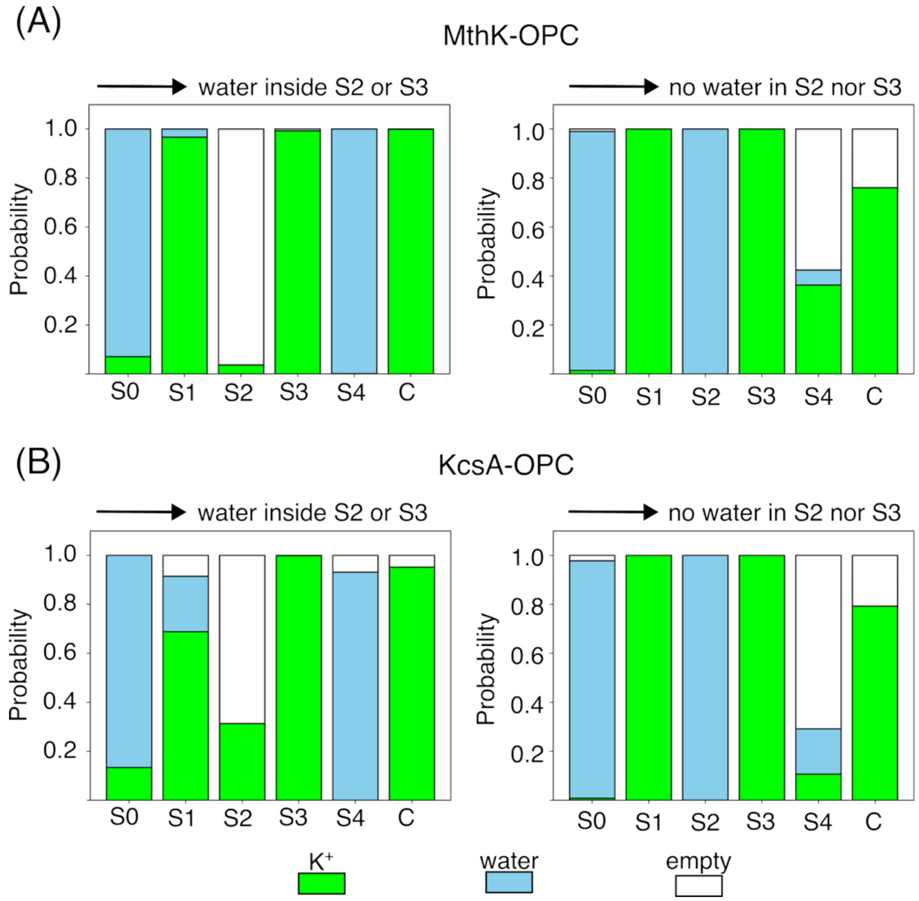

Figure S5. Average state of the SF that precedes the entrance of water molecules in S2 or S3 (left) or the depletion of S2 and S3 from water molecules (right). The average state of the SF that leads to the entrance of water molecules in S2 or S3 was calculated considering all the microscopic states of the MSM with no water in S2 nor S3 (*source*) that are connected to any microscopic state with water molecules in S2 or S2 (*sink*). Then, the weighted average of the probability of the binding site being empty, occupied by water, or occupied by potassium ions were calculated. The same method, switching *source* with *sink*, was used to calculate the average state of the SF that leads to the depletion of S2 and S3 from water molecules. Data refers to simulations of the MthK/KcsA channel with the OPC water model at a membrane potential of 400 mV.
